# The response clamp: functional characterization of neural systems using closed-loop control

**DOI:** 10.3389/fncir.2013.00005

**Published:** 2013-01-30

**Authors:** Avner Wallach

**Affiliations:** Department of Neurobiology, Weizmann Institute of ScienceRehovot, Israel

**Keywords:** response clamp, control, closed-loop, physiology, psychophysics

## Abstract

The voltage clamp method, pioneered by Hodgkin, Huxley, and Katz, laid the foundations to neurophysiological research. Its core rationale is the use of closed-loop control as a tool for system characterization. A recently introduced method, the *response clamp*, extends the voltage clamp rationale to the functional, phenomenological level. The method consists of on-line estimation of a response variable of interest (e.g., the probability of response or its latency) and a simple feedback control mechanism designed to tightly converge this variable toward a desired trajectory. In the present contribution I offer a perspective on this novel method and its applications in the broader context of system identification and characterization. First, I demonstrate how internal state variables are exposed using the method, and how the use of several controllers may allow for a detailed, multi-variable characterization of the system. Second, I discuss three different categories of applications of the method: (1) exploration of intrinsically generated dynamics, (2) exploration of extrinsically generated dynamics, and (3) generation of input–output trajectories. The relation of these categories to similar uses in the voltage clamp and other techniques is also discussed. Finally, I discuss the method's limitations, as well as its possible synthesis with existing complementary approaches.

## Motivation

A paramount goal of any neurophysiological study is to identify and characterize the function of neural systems. What kind of methodology can one employ in order to achieve this goal? A compelling option is to use the framework of control theory and signal processing, which engineers utilize to characterize artificial systems. The first step in this methodology is to define the system's input and output variables. Then, a set of signals is selected (e.g., step, pulse, or harmonic functions) and is applied to the system's input, while the output signal corresponding to each input is observed. Finally, the system is characterized in terms of its *input–output relations*, namely the conversion laws that dictate what kind of output arises in response to any given input (including novel, untested stimuli). Another realization of this approach is to use *noise* as input and to deduce the input–output relations using reverse correlations. This “open-loop” methodology is very efficient when simple systems are involved: a linear time-invariant (LTI) system (e.g., a classical resistor-capacitor circuit) may be fully characterized-based solely on its response to a single step function; simple non-linear elements, such as analog transistors in their “linear” regime, may also be studied using “small signal” (i.e., harmonic) analysis.

The application of such tools to biological systems, however, is severely limited. First, these systems are invariably composed of non-linear elements which exhibit sharp threshold phenomena, i.e., small changes in their input may cause abrupt and significant changes in their output. Second, biological systems are stochastic, with a response variance which is often comparable in magnitude to the response mean (Arieli et al., 1996). Finally, time and activity-dependent processes continuously change the properties of the system; such changes are referred to as inactivation, adaptation, habituation, learning, etc. Therefore, the history of activity impacts on the system's internal variables, which in turn affect future activity—and so forth. This internal feedback results in dynamic instabilities that are manifested in complex trajectories of the system's output.

This challenge was confronted by Hodgkin et al. (1952) in their analysis of the mechanisms underlying the generation of action-potentials. There, too, the dynamics of non-linear and history-dependent internal variables (in this case, membrane conductances) result in a complex voltage trajectory. The breakthrough in that study came with the development of a closed-loop technique called the *voltage clamp*, in which the system's output is stabilized by applying feedback control. Once the voltage is controlled, the dynamics of the membrane conductances were significantly simplified and could be measured by analyzing the control signal (i.e., the feedback current). This enabled comprehensive study and quantitative modeling of the system.

The essence of the clamp rationale, therefore, is to use control as a tool for system characterization; it inverts the experimental approach, determining the output of the system and observing the input signal required in order to obtain this desired output. One might expect this inverted system to simply reflect the behavior of the open-loop (i.e., current clamp) scenario, yet this is seldom the case in the non-linear, time-variant systems ubiquitous in physiology. The voltage clamp and other methods that emanated from it were extremely instrumental in elucidating the mechanisms of excitability. They did not, however, directly target the functionality of neural systems beyond the molecular level as the object of control.

The rationale of the voltage clamp technique was generalized to the study of neural systems at the functional, phenomenological level in a recently introduced method called the *response clamp* (Wallach et al., 2011). The current contribution aims at offering a comprehensive perspective of this method, its possible applications and extensions, as well as of its limitations. Note that, while termed a Review, this article does not attempt to provide an expansive outlook on the field of closed-loop methodology (for such a review of closed-loop physiology, for instance, see Arsiero et al., 2007).

## The response clamp exposes functional state variables of neural systems

The response clamp method utilizes a simple control procedure which allows robust manipulation of the system's response dynamics. First, a selected response feature is either directly measured or estimated from the system's activity. Then, a *Proportional-Integral-Derivative* (PID) controller (Levine et al., 1996) adjusts a stimulation parameter related to that feature in close-loop, so that the system's behavior converges to some desired pattern. The procedure eliminates the feedback from the system's response dynamics to its internal state dynamics. Moreover, these internal (and otherwise hidden) state dynamics are exposed to continuous measurement by analysis of the control signal. To demonstrate this let us use the example of clamping the response *probability*, which served in previously published studies (Marom and Wallach, 2011; Wallach and Marom, 2012).

**Figure 1:**
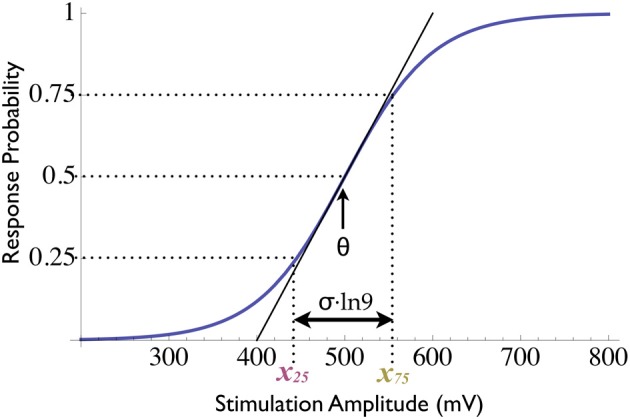
**Sigmoidal input–output relations.** The response probability's dependence on stimulation intensity follows a sigmoidal function with two parameters (state variables): the threshold θ and the dynamic range σ (see Equation 1). When two response clamps are used, one controller may clamp to 0.75 and the other to 0.25, thus yielding measurements of two distinct loci on the response curve (denoted *x*_75_ and *x*_25_, respectively). The mean of these measurements is the threshold, while their difference is proportional to the dynamic range.

Many excitable systems are characterized by an “all-or-none” response to external perturbation. While the responses themselves, once evoked, are stereotypical and uniform, the *probability* of evoking these responses is graded and depends on various stimulation parameters, as well as on the present state of the system. Some qualitative understanding of this dependence is required in order to establish control over the response probability; the easiest case is when the probability is monotonically related to some stimulus feature (e.g., intensity or contrast relative to the background). The most abundant form of such monotonic relationships in physiology and psychophysics is the *sigmoidal curve*, which exhibits threshold and saturation phenomena; several mathematical functions were used to model such sigmoidal relations, e.g., the *error function*, the *hyperbolic tangent* and the *logistic curve*. Let us consider the latter, which is characterized by a *threshold* parameter θ and a *dynamic range* parameter σ,

(1) $$P(x;t)=\frac{1}{1+e^{\;-\;\frac{\left(x(t)\;-\;\theta (t)\right)}{\sigma (t)}}},$$

where *P* is the response probability and *x* is the stimulation intensity (Figure 1). Note that small values of σ signify a steep sigmoid and therefore high sensitivity to changes in stimulation intensity [the maximal slope being (4σ)^−1^].

Due to the monotonic nature of this relationship, the response probability may be controlled by continuously adjusting the stimulation intensity using a negative feedback loop; the PID algorithm of the response clamp is a simple and efficient way to realize this loop. If we choose 50% as our target response probability, it is readily apparent that the stimulation sequence produced by the controller must satisfy at all times *x*(*t*) = θ(*t*), i.e., the control signal in fact reflects the instantaneous threshold, a key functional state variable of the system. In practice this measurement contains some degree of inherent noise, since the system is stochastic and the response probability must be estimated using a finite number of samples.

Thus, using one response clamp controller, one locus on the input–output curve is tracked, providing a single-parameter characterization of these relations. A more detailed characterization is possible using multiple controllers, each clamping to a different value. The controllers take turns in stimulating the system (i.e., they are “time-multiplexed,”) each using only the responses to its own stimuli in the control algorithm. This configuration provides non-simultaneous, mutually independent measurements of the system [see Wallach and Marom (2012) for details]. Thus, a multiple clamp set-up consisting of *n* controllers tracks *n* points in the input–output curve. Producing the state variables of interest from this set of measurements might require some “coordinate transform,”

(2) $$\vec{S}=f\left(\vec{X}\right),$$

where $\vec{X}$ is the vector of measurements (i.e., the set of *n* control signals), *f* is some function and $\vec{S}$ is a vector of *m* state variables of interest (*m* ≤ n). If, for example, the transform is linear, then

(3) $$\vec{S}=T\cdot \vec{X},$$

where ***T*** is some *m* × *n* matrix.

In the example of the sigmoid relations presented in Equation (1), for instance, using two controllers enables tracking both the threshold and the dynamic range variables: the two controllers are used alternatingly, one clamps to 25% while the other to 75% response probability. Thus, the overall response is clamped to a constant 50% of the total stimulation and two distinct loci on the response curve, denoted *x*_25_ and *x*_75_, are measured (see Figure 1). The two state variables θ(*t*) and σ(*t*) are produced using the linear mapping^1^

(4) $$\theta (t)=\frac{x_{75}(t)+x_{25}(t)}{2}\;\sigma (t)=\frac{x_{75}(t)-x_{25}(t)}{\ln9}.$$

**Figure 2:**
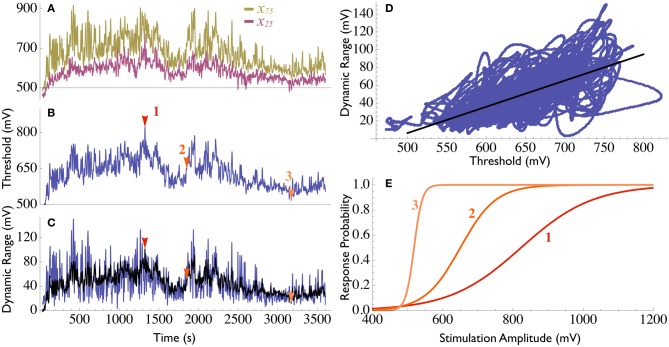
**Neuronal threshold and range dynamics. (A)** Measurement of *x*_75_ (yellow) and *x*_25_ (purple) during 1 h of double clamping an isolated neuron *in vitro* (see Methods in Wallach et al., 2011). The two measurements are highly correlated. The neuronal threshold θ **(B)** and the dynamic range σ **(C)**, were computed using Equation (4) (blue line in both). **(D)** When the measurements are displayed in the threshold/range state plane, the significant correlations between them is evident. Fitting with a linear relation [Equation 5, black line in panel **(D)**, *R*^2^ = 0.52] enables the estimation of the dynamic range based on the instantaneous threshold [black line in panel **(C)**]. **(E)** Examples to the instantaneous I/O relations (Equation 6) at three different points in time [marked with colored arrowheads in panels **(B)** and **(C)**]. The curve becomes stretched as the threshold increases. Note that as the threshold approaches the minimal value θ_0_, the curve approaches a step-function, i.e., the neuron becomes a deterministic element.

Figure 2A demonstrates typical recordings obtained using the double clamp procedure on isolated neurons *in vitro*. The state variables θ(*t*) and σ(*t*) (in this case they are both expressed in mV), obtained using Equation (4), are presented in Figures 2B,C.

The use of multiple clamps, therefore, allows for a detailed, multiple-variable characterization of the system. This comes at the price of decreasing the temporal resolution of the measurements, since the total stimulation rate must be distributed between a number of controllers. Finally, multiple clamps may also be used to study structured or modular systems, e.g., the dynamics of coupling between interrelated systems or the integration of different inputs within the same system.

## Applications of the response clamp

The possible applications of the method may be classified into three categories: (1) exploration of intrinsically generated dynamics (2) exploration of extrinsically generated dynamics, and (3) generation of input–output trajectories.

### Exploring intrinsically generated dynamics

Let us return once more to our main source of inspiration, the voltage clamp technique. The studies pioneering this method, and many that followed, applied it on isolated systems (e.g., the giant squid axon) to observe the internal dynamics of ionic conductances at different voltage levels. Later, current fluctuations in microscopic, voltage-clamped membrane patches were analyzed to study the same issue at the molecular level, a technique termed “patch clamp” (Neher et al., 1978). In both cases, therefore, the voltage clamp was used to study the dynamics of the state variables with relations to changes in the clamped variable itself. Let us call such dynamics “intrinsic,” as they are not related to some event occurring outside of the clamped system.

Similarly, the response clamp may be used to study the intrinsic dynamics of a system's state variables at the functional level. Such was the application of the response clamp in behavioral psychophysics (Marom and Wallach, 2011), where the subjects' response fluctuations in the clamped and unclamped scenarios were compared. The results suggest that these fluctuations are markedly restrained in closed-loop conditions, namely when the subject's actions have some (unconscious) effect on future stimuli. This led the authors to postulate that the well-documented trial-to-trial variability does not reflect, as previously suggested, an intrinsic “noise” process; rather, it stems from the unnatural open-loop experimental paradigm. The response clamp may be used in a similar manner to investigate the relations between psychophysical dynamics and brain activity (Monto et al., 2008), or to study the factors driving threshold fluctuations at the cellular, synaptic or network levels.

To further demonstrate how closed-loop analyses provide new insights into functional properties of a system, let us apply the double response probability clamp experiment discussed above to study intrinsic response dynamics of isolated neurons (Gal et al., 2010). By plotting the two derived state variables, θ and σ, against each other (Figure 2D), it becomes evident that the long term fluctuations of the dynamic range are highly correlated with those of the threshold. The relations between these two variables may thus be approximated using a linear expression, namely,

(5) $$\sigma (t)=\alpha \left(\theta (t)-\theta_{0}\right).$$

Equation (5) may be used to produce a smoothed estimate of σ based on the values of θ (black line in Figure 2C). The instantaneous input–output relations are therefore simplified in this case to a single state variable expression,

(6) $$P(\bar{x},\bar{\theta }(t))=\frac{1}{1+e^{\;-\;\left(\bar{x}\;-\;\bar{\theta }(t)\right)/\alpha \bar{\theta }(t)}},$$

where $\bar{x}=x-\theta_{0}$ and $\bar{\theta }=\theta -\theta_{0}$ are the *relative* stimulation amplitude and threshold, respectively. Figure 2E visualizes such instantaneous I/O relations in three different instants during the recording (marked with arrowheads in Figures 2B,C). This result is, in fact, quite expected, as scaling of sensitivity to changes with stimulation magnitude is a ubiquitous phenomenon in both physiology (Abbott et al., 1997) and psychophysics (e.g., the Weber–Fechner law, see Carterette and Friedman, 1974). Note that the offset parameter θ_0_ has a biophysical significance: it is the threshold value at which the dynamic range becomes zero, i.e., the neuron becomes *deterministic* (the I/O relations become a step function). θ_0_, therefore, is the minimal stimulation amplitude required to generate a spike at the maximal excitable state of the neuron, constituting an example to how analyses of intrinsic fluctuations of the system's state variables (reflected in the response clamps' control signals) yield novel findings as to the functional properties of the system.

### Exploring extrinsically generated dynamics

While much can be learned by studying isolated systems, neural systems are invariably embedded in networks and environments, where they interact with many external factors; any neuron, for instance, is affected by the activity of its peers via synaptic inputs converging onto it. The voltage clamp proved very beneficial in investigating the mechanisms of this communication by providing measurements of the *post synaptic currents* (Hagiwara and Tasaki, 1958): the membrane potential is held constant at some desired value, and changes in the feedback current *due to an external event* (e.g., an action potential generated in a neighboring cell) are measured. Using this application of the clamp, one may isolate individual input components (i.e., by clamping to a specific reversal potential) and separate them from the dynamics of the system itself (by preventing the generation of action potentials).

Similarly, the response clamp may be used to study changes in the functional behavior of systems due to interactions with their external environment. In a recently published paper (Wallach and Marom, 2012), the long-term effects of network events (brief episodes of synchronous, network-wide activity, also called “bursts” or “population spikes”) on neuronal threshold were analyzed. Since the measurements are inherently noisy the effect of a single event was usually too small to observe and event-triggered averaging was applied. Using this procedure it was shown that network synchronous events induce a long lasting, bi-phasic deflection of the neuronal threshold. The results demonstrate interrelations between the dynamics at the two levels: the magnitude of the network event is reflected in the amplitude of the neuronal threshold deflection, while the relaxation of the threshold back to baseline is correlated with the recovery dynamics of network excitability.

These results demonstrate how the response clamp could be applied to the study of such extrinsically generated dynamics. Any measurable external influence on the clamped system (either subject to experimental control or autonomous) may be analyzed in a similar manner; the effects of various chemical compounds (such as neuromodulators or toxins) on overall cellular excitability, for instance, may be thus quantified. Similarly, the method may be implemented to study the interactions between different inputs to the same system: the response to one source may be clamped, and changes in the control signal due to activation of the second source may be recorded.

### Generating input–output trajectories

Like many other closed-loop stimulation techniques (e.g., Wagenaar et al., 2005; Arsiero et al., 2007; Rolston et al., 2010), the response clamp offers the capability to control the activity patterns of neural systems. This capability may, in and by itself, be useful in different experimental scenarios. In such cases, the control signal is not used for analysis; rather, the effect of the produced dynamics on other (non-clamped) variables is explored. The most notable derivative of the voltage clamp technique in this context is the *dynamic clamp* (Sharp et al., 1993), in which the current injected in a closed-loop effectively adds or removes conductance components to the cell; the contribution of these conductances to the overall system behavior, and not the injected current, is the subject of analysis in this method.

In the response clamp, it is this “overall system behavior” that is manipulated. For instance, let us assume that an isolated system is repetitively stimulated at rate *f*_in_, and the response probability to this stimulation is clamped to some value *p*. The activity rate of this system, *f*_out_, is therefore also clamped, since

(7) $$f_{\text{out}}=f_{\text{in}}\cdot p.$$

Thus, by maintaining *f*_in_ constant and varying *p*, one may precisely produce desired activity patterns (see Figure 5 in Wallach et al., 2011). This may be useful if the clamped system serves as an input stage for downstream systems. Interestingly, Toettcher et al. (2011) recently suggested a similar approach (also using a PID-based algorithm) to control the dynamics of intracellular signaling pathways, thus generating a well-controlled, repeatable input to downstream components in the pathway.

Yet one may use this tool to do more than just control the output dynamics: by controlling both the clamped response and the input, regions of the *input–output space* may be efficiently covered. For instance, by jointly altering *f*_in_ and *p* (in opposite directions), one may observe the system's behavior at different input levels, while maintaining the output level (*f*_out_) constant. Exploring various input–output combinations may elucidate the contributions of input-dependent effects (i.e., direct effects of stimulation) and activity-dependent effects to the overall behavior.

## Limitations of the response clamp and comparison with other approaches

### Coverage of the time-scale spectrum

The voltage clamp served as a source of inspiration and as a reference methodology for the development of the response clamp and its applications. However, a different range of time-scales is accessible in each of these two techniques. In voltage clamp, the controller (the feedback amplifier) is both extremely fast (i.e., its time-constant is much shorter than that of the clamped membrane) and powerful (i.e., the feedback gain is high) so that the clamp process is, for all practical purposes, instantaneous. Thus, the voltage clamp enables the investigation of even the fastest processes in the membrane (e.g., fast activation of sodium channels). Application of the voltage clamp to the study of extremely slow processes, however, is limited in several respects. First, voltage clamp is presently performed using physically invasive intracellular electrodes, a procedure which sets a practical upper bound to the duration of recordings. This technical limitation may be theoretically circumvented if a non-invasive realization of the method is invented (e.g., by harnessing optical techniques for both voltage measurements and current injection). However, voltage clamp is “invasive” in a different, more fundamental sense: as long as the cell is clamped, its natural behavior (i.e., emitting action-potentials) is completely shut-down. Thus, even if a non-invasive voltage clamp did exist, the results obtained using this technique would have little to do with natural long term dynamics of excitability.

The response clamp provides access to a range of time-scales which is complementary to those covered by the voltage clamp. On one hand, access to the very fast time-scales may be limited due to stimulation constraints (e.g., maximal possible stimulation intensity or rate) and to the time-scale of the control algorithm (determined by the various control parameters). On the other hand, the straightforward realization of the method using non-invasive means of stimulation and recording (e.g., extracellular electrodes), and the fact that the cell's natural spiking behavior remains intact, extends the experimental access into extremely long-term processes. By determining the time-scale of the clamped dynamics, the response clamp provides an experimental tool to separate processes of different time-scales governing the behavior.

### Applicability to controllable systems

A prerequisite to any application of the response clamp is to establish reliable control of the response feature of interest by manipulation of some input parameter. In the systems studied so far, establishing this control was particularly straightforward since the input–output relations were monotonically non-decreasing (e.g., the sigmoidal curve in Equation 1). In systems where these relations are of a more complex nature (e.g., bell shaped or multi-modal), a more elaborate control algorithm is required (Astrom and Wittenmark, 1994). Moreover, in some systems the relevant input feature (the so called “receptive field”) may be unknown. In such cases, some algorithm that finds this relevant input feature within the space of all possible inputs must be instated, in order for the clamp to be applicable. Such a combined solution is discussed in the next section.

### Reverse correlation and white-noise analyses

The response clamp demonstrates how closed-loop control may be used as a tool for system characterization. An important open-loop alternative which was already mentioned above is the *reverse correlation* approach. In this method the input–output relations of a system are exposed by computing various weighted statistics of the input variable, with the assigned weights derived from the output variable. The most common of these techniques is the *Spike-Triggered Average* (STA) and its extensions, which were used extensively in order to estimate the receptive fields of various neurons [see Simoncelli et al. (2004), and references therein]. When the input is under experimental control, approximated white-noise is usually applied, so that equal energy is applied across a broad range of time-scales (alternatively, whitening procedures may be used). This was shown to guarantee (under some additional restrictive conditions, see Paninski, 2003) that the estimation is unbiased.

There are several limitations to the use of reverse correlation methods. First, if the stimuli space is multi-dimensional, unbiased coverage of this space is very difficult experimentally, as the number of stimuli needed increase exponentially with each additional dimension [Benda et al. (2007) already purposed closed-loop stimulation as a method to efficiently sample this space when systematic, open-loop coverage is impractical]. More importantly, the underlying (and often unstated) assumption in these methods is that the system is feed-forward and static; the receptive field derived using STA is tightly related to the linear stage of *Linear-Nonlinear-Poisson* neuronal models, which do not account for refractoriness, output-dependent processes or threshold dynamics. Moreover, since the space of all possible output dynamics is not necessarily covered (e.g., high firing rates are rarely reached), such output-dependent effects may not be fully expressed in white-noise perturbation.

White-noise analysis, however, holds the considerable advantage of enabling identification of the input features to which the system is sensitive using very limited *a-priori* knowledge (e.g., the relevant modality). As it happens, this advantage precisely addresses the above mentioned impediment to the implementation of the response clamp, namely the need to identify a relevant and effective control variable. Thus, STA and the response clamp may be used in tandem, each method complementing the other: first, STA is implemented to expose the “static” or “baseline” receptive field; then, the response clamp uses this receptive field to produce the control variable, in order to expose the dynamic and output-dependent processes of the system.

### Comparison with other closed-loop techniques

Closed-loop control is, in itself, a widely accepted tool in many fields of research. Physiologists have employed closed-loop techniques and protocols to control aspects of neuronal activity at all levels of biological organization [see Arsiero et al. (2007) and references therein]. Already in the late 1960's, Eberhard E. Fetz showed that the activity of a single cortical neuron may be reinforced by applying closed-loop control of food pellets delivery (Fetz, 1969).

In psychophysics, a variety of procedures were developed over the past few decades in order to measure the psychometric threshold in closed-loop (Treutwein, 1995). The underlying assumption in all these procedures is that, in a given experiment, the threshold is *static*, and hence the procedure is stopped once it “converges” to a reliable estimate of this threshold. The key novelty in applying the response clamp to psychophysical investigations (Marom and Wallach, 2011), therefore, is in the analysis of post-convergence fluctuations of the threshold.

The fundamental difference between both the voltage- and response-clamp methods and other closed-loop techniques is that the control signal in all these techniques (be it food-pellet delivery rate, stimulation amplitude, etc.) is seldom used in order to gain access to the dynamics of hidden state variables. An interesting exception worth mentioning is a clinical method called *glucose clamp* (DeFronzo et al., 1979), developed in order to diagnose insulin secretion and resistance by analysis of the control signals (rates of glucose/insulin perfusion or infusion). This method, though rarely used in clinical practice, is considered the “gold standard” in the diagnosis of diabetes.

It should be stressed that the use of the PID control algorithm is, in and by itself, of no fundamental importance to the realization of the response clamp. This algorithm was chosen owing to its simplicity and generality; the PID is a pure-feedback, *model free* algorithm, and therefore little *a-priori* knowledge of the controlled system is required in order to implement it. Any other algorithm which efficiently clamps the system's response may be used. One might expect, for instance, that using other adaptive psychophysical protocols would yield similar results to those of Marom and Wallach (2011); rigorous examination of this prediction, however, is yet to be performed.

## Concluding remarks

The voltage clamp revolutionized the way physiologists study the mechanisms of excitability and synaptic communication. The response clamp method extends the clamp rationale toward functional characterization of neural systems. It offers a general framework for closed-loop exploration that may be implemented at any level of organization, using any available technique of measurement or perturbation. It may also be combined with complementary, open-loop approaches such as white-noise analysis. Finally, one may envision a paradigm in which voltage- or dynamic-clamp “command” is controlled in closed-loop by the response clamp algorithm. Such a multi-layered clamp set-up may aid in bridging the gap between mechanistic and functional characterization of neural systems.

### Conflict of interest statement

The author declares that the research was conducted in the absence of any commercial or financial relationships that could be construed as a potential conflict of interest.

## Footnotes

^1^This is a two-dimensional case of Equation (3), where $\vec{X}=\left(\begin{array}{c} x_{75}\\ x_{25} \end{array}\right)\text{​}$, $\vec{S}=\left(\begin{array}{c} \theta \\ \sigma \end{array}\right)$ and $T=\left(\begin{array}{cc} 0.5 & 0.5\\ {\left(\ln9\right)}^{-1} & -{\left(\ln9\right)}^{-1} \end{array}\right)$
